# Systematic review of Integrated Disease Surveillance and Response (IDSR) implementation in the African region

**DOI:** 10.1371/journal.pone.0245457

**Published:** 2021-02-25

**Authors:** Caitlin M. Wolfe, Esther L. Hamblion, Emmanuel K. Dzotsi, Franck Mboussou, Isabelle Eckerle, Antoine Flahault, Claudia T. Codeço, Jaime Corvin, Janice C. Zgibor, Olivia Keiser, Benido Impouma

**Affiliations:** 1 Health Emergency Information and Risk Assessment, Health Emergencies Programme, World Health Organization Regional Office for Africa, Brazzaville, Republic of Congo; 2 University of South Florida College of Public Health, Tampa, Florida, United States of America; 3 Division of Infectious Diseases, Geneva Centre for Emerging Viral Diseases, University Hospital of Geneva, Geneva, Switzerland; 4 Institute of Global Health, University of Geneva, Geneva, Switzerland; 5 National School of Public Health (ENSP/Fiocruz), Fundação Oswaldo Cruz (FIOCRUZ), Rio de Janeiro, Brazil; Helen Keller International, SIERRA LEONE

## Abstract

**Background:**

The WHO African region frequently experiences outbreaks and epidemics of infectious diseases often exacerbated by weak health systems and infrastructure, late detection, and ineffective outbreak response. To address this, the WHO Regional Office for Africa developed and began implementing the Integrated Disease Surveillance and Response strategy in 1998.

**Objectives:**

This systematic review aims to document the identified successes and challenges surrounding the implementation of IDSR in the region available in published literature to highlight areas for prioritization, further research, and to inform further strengthening of IDSR implementation.

**Methods:**

A systematic review of peer-reviewed literature published in English and French from 1 July 2012 to 13 November 2019 was conducted using PubMed and Web of Science. Included articles focused on the WHO African region and discussed the use of IDSR strategies and implementation, assessment of IDSR strategies, or surveillance of diseases covered in the IDSR framework. Data were analyzed descriptively using Microsoft Excel and Tableau Desktop 2019.

**Results:**

The number of peer-reviewed articles discussing IDSR remained low, with 47 included articles focused on 17 countries and regional level systems. Most commonly discussed topics were data reporting (n = 39) and challenges with IDSR implementation (n = 38). Barriers to effective implementation were identified across all IDSR core and support functions assessed in this review: priority disease detection; data reporting, management, and analysis; information dissemination; laboratory functionality; and staff training. Successful implementation was noted where existing surveillance systems and infrastructure were utilized and streamlined with efforts to increase access to healthcare.

**Conclusions and implications of findings:**

These findings highlighted areas where IDSR is performing well and where implementation remains weak. While challenges related to IDSR implementation since the first edition of the technical guidelines were released are not novel, adequately addressing them requires sustained investments in stronger national public health capabilities, infrastructure, and surveillance processes.

## Introduction

The countries in the WHO African region frequently experience outbreaks and infectious disease epidemics, resulting in large-scale morbidity, disability, and deaths. The scale of impact is exacerbated by a lack of robust health systems and health infrastructure, including weak surveillance, preparedness and response systems leading to late detection and ineffective response to these outbreaks. To address this situation, the World Health Organization (WHO) Regional Office for Africa (AFRO) developed the first technical guideline (TG) and began to implement the Integrated Disease Surveillance and Response (IDSR) strategy in 1998 [1]. While focused at the district level, the goal of the IDSR strategy is to develop sufficient surveillance and response capacities at each level of the national health system to produce a flexible priority disease surveillance system [1–5]. The 1998 IDSR TG targeted nineteen priority communicable diseases, divided into four categories: epidemic-prone diseases, diseases targeted for eradication, diseases targeted for elimination, and diseases that were endemic [1].

Since the initial implementation, social, economic, environmental, and technical changes have altered the health landscape in the region. The emergence of new diseases, conditions, and events necessitated the review of public health priorities for surveillance and response. While the initial goals of IDSR focused on communicable diseases, increased incidence of non-communicable diseases in the region required their inclusion in the IDSR strategy. Additionally, the emergence of pandemic and pandemic-potential influenza (H1N1 and avian) emphasized the importance of community surveillance for linking detection to rapid confirmation and response [6]. The adoption of the revised International Health Regulations (IHR) in 2005 further demanded a revision of the 1998 IDSR TG in 2010 [2]. This revision proposed an alteration to the four categories to: epidemic prone diseases, diseases targeted for eradication or elimination, other major diseases, events, or conditions of public health importance, and diseases or events of international concern [2, 7] (S1 Appendix).

The 2010 revision outlined core steps necessary for effective surveillance systems [1]. These functions included (1) using standardized case definitions to identify priority diseases, conditions, and events; (2) reporting of suspected cases, conditions, or events to the next level; (3) analyzing collected data for trends and interpreting the findings; (4) investigating and confirming suspected cases, outbreaks, or events; (5) preparing in advance of outbreaks or public health events to enable immediate response actions; (6) implementing an appropriate public health response; (7) providing feedback across levels of the surveillance system; and (8) continually evaluating and improving the system. More broadly, the IDSR strategy requires the correct use of case definitions, laboratory confirmation, data analysis, interpretation of findings, and reporting [2].

The status of IDSR implementation has differed by country across the region. In 2012, Phalkey et al. [8] conducted a systematic review of the literature, assessing implementation challenges with the IDSR strategy and documenting lessons learned in sixteen countries in the African region. This review concluded that challenges with IDSR implementation are systemic in nature, emphasized the benefits of skill-based training for personnel, and strengthening of the support to surveillance functions alongside health care infrastructures at the district level [8].

Since then, the international community witnessed the importance of a strong and functional IDSR strategy in the wake of recent outbreaks. Robust surveillance and response capacity, along with trained and informed personnel, are hallmarks of a strong public health system. The consequences of delayed action to infectious disease outbreaks, as clearly demonstrated during the West Africa Ebola outbreak [9, 10], can be devastating and are detrimental to national and regional economies. The ability to respond efficiently and effectively can help mitigate their devastation. One of the key requirements of IDSR is the development and dissemination of information products, which include the writing and publication of scientific articles on IDSR, to inform decision-making by policy makers. However, peer-reviewed scientific articles as IDSR information products are still rare. Reviewing the published literature, particularly those focusing on recent disease outbreaks, can help to identify areas where IDSR implementation is performing well and where implementation remains weak, further highlighting areas for research and prioritization.

As the health situation in the WHO African region continues to evolve, a third revision of the 2010 IDSR TG occurred in 2019 [11] (S2 Appendix). This revision aimed to align with the introduction of the Regional Strategy for Health Security and Emergencies 2016–2020 in the WHO African region [12], and utilizes the opportunities offered by new information technologies such as mobile phone networks, increased broadband internet connectivity and electronic surveillance systems. Prior to the revision, ongoing efforts were underway to further strengthen IDSR in the region. In late April 2018, the WHO AFRO Health Information and Management (HIM) team hosted representatives from 10 countries at an induction meeting in Dakar, Senegal to initiate in-country missions to strengthen the management and use of data on IDSR priority diseases. The in-country missions, conducted by WHO AFRO consultants, took place over the two to three months immediately following the induction meeting. While the in-country missions focused on management and use of IDSR data, the need to examine the representation of IDSR implementation in scientific articles also emerged. As such, a systematic review of IDSR implementation in published literature was conducted using PubMed in June 2018, building on the findings from Phalkey et al. [8] in 2012. After subsequent outbreaks in the region the following year, the search in PubMed was conducted again to capture any articles published from June 2018 through November 2019. To ensure the systematic review was thorough, Web of Science was also searched for the same date parameters (July 2012 through November 2019).

The objective of this analysis was to systematically review and document the peer-reviewed lessons learned and challenges identified surrounding the implementation of IDSR in the WHO African region and facilitate the identification of common barriers and areas for future research and prioritization. This analysis incorporates recent research and health systems strengthening efforts in the region to further strengthen the findings of the previous review [8]. Assessing the capacities of the core and support IDSR functions will also identify components of the technical guidelines where more in-depth training is required in Member States.

## Methods

### Search strategy

In accordance with the PRISMA 2009 guidelines [13], we conducted a systematic review of the peer-reviewed literature published from 1 July 2012 to the 13 November 2019 in three phases. The first phase consisted of a search conducted in June 2018 that identified relevant literature indexed in PubMed published between 1 July 2012 through 20 June 2018. As mentioned previously, following subsequent outbreaks in the region the following year, the search in PubMed was conducted again to capture any articles published from 30 June 2018 through 13 November 2019. To ensure the systematic review was thorough, a third search was performed in Web of Science using the same criteria and covering the same time period (1 July 2012–13 November 2019). Targeted search strategies were constructed for PubMed and Web of Science in both English and French (S3 Appendix). Key search terms included "Integrated Disease Surveillance and Response," “IDSR,” "IDSR Implementation," or "IDSR Evaluation" and the French equivalents in the title and abstract of indexed literature. We reviewed the titles and abstracts using the inclusion and exclusion criteria outlined below. Full-text articles of included titles and abstracts were downloaded and reviewed for data extraction. In addition, we examined the references of included studies.

Included literature consisted of peer-reviewed, full-text articles that discussed the use of IDSR strategies and its implementation, assessment of IDSR implementation or strategies, or articles discussing surveillance of diseases covered in the IDSR framework. Only articles focusing on countries in the WHO African region were included. Results published only as abstracts or presented in conferences without full accompanying full-text publications were excluded, as were previous systematic reviews of IDSR implementation to capture the most recent available research. Articles that discussed diseases covered in the IDSR framework but did not relate their assessment or findings back to the IDSR strategy were also excluded.

### Data extraction and analysis

A pre-determined extraction form with defined variables was used to extract data from each article. Data were extracted by one reviewer (CMW) first, and once the initial extraction was completed, the extracted data as well as rationale for excluded articles was reviewed by the second reviewer (ELH). Any discrepancies between the two reviewers were resolved by consensus. Articles were listed and summarized by core and support functions, key outcomes, and gaps requiring further studies. Core IDSR functions include the detection and notification of priority diseases; laboratory confirmation; data reporting; data management; data analysis; preparedness and response; and information dissemination. Support functions include training, supervision, and resources, grouped together under the larger “personnel” umbrella. Extracted information included the location of the study, year of IDSR implementation (for specific countries), year of publication, level of focus (national, district, health facility), disease focus, IDSR core and support functions, challenges with IDSR implementation, identified gaps requiring further study, and key recommendations. The extracted data was summarized and analyzed to report descriptive statistics using Microsoft Excel and Tableau Desktop 2019.

### Funding

There was no specific funding source for this study, however author O. Keiser was supported by a grant from the Swiss National Science Foundation (no. 163878) during the revision process.

### Protocol

No existing protocol was used for this review.

## Results

**Fig 1:**
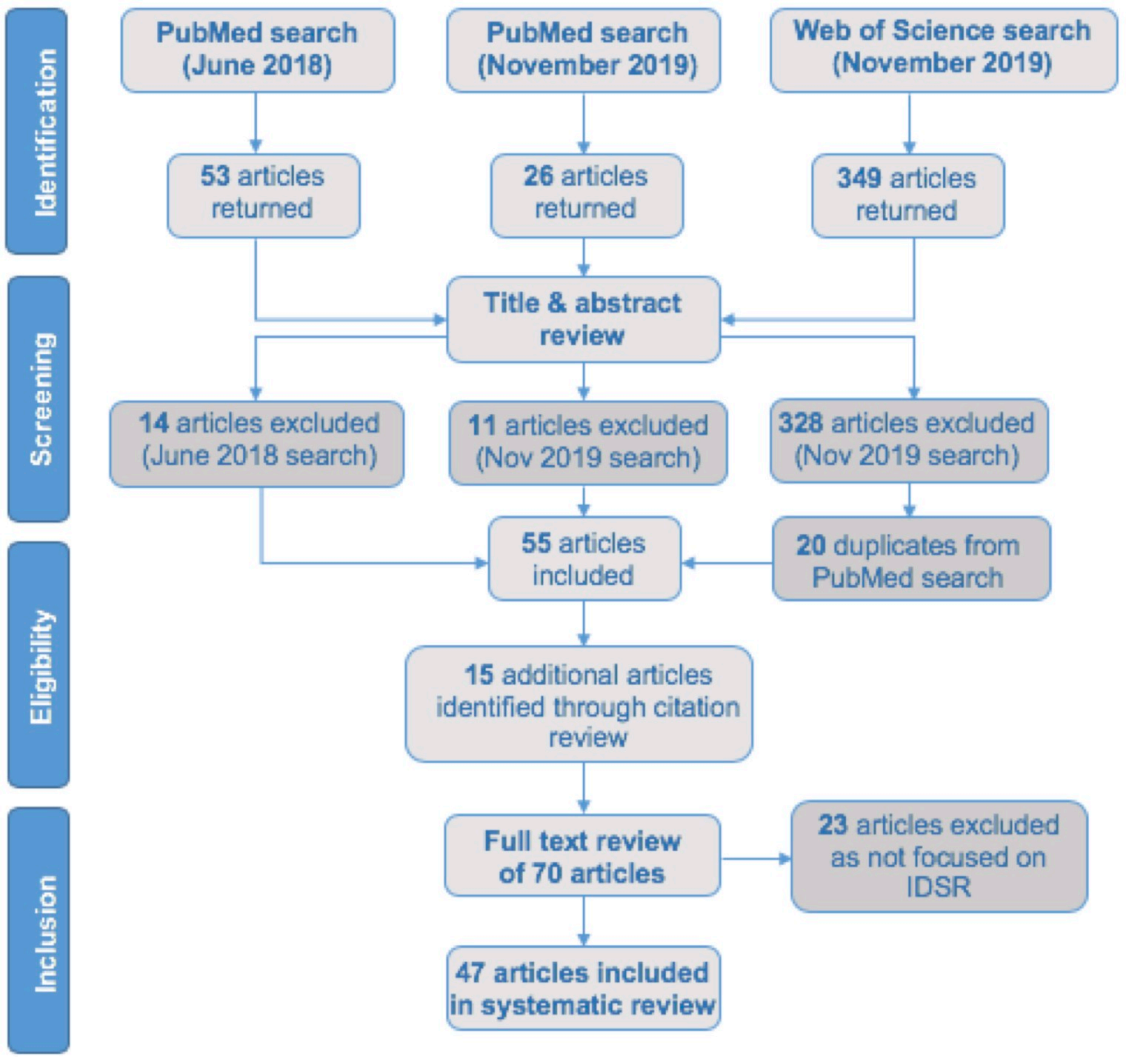
Study selection (PRISMA diagram). PRIMSA diagram of the selection process for articles included in this review.

The search strategy returned 79 results within PubMed and 349 results within Web of Science. After reviewing the title and abstract of each article, 54 were included for full-text review from PubMed. Of the 21 articles that were included for full-text review from Web of Science, 20 were duplicates of those included from the PubMed search. During the full text review, an additional 15 were identified through the references in the returned articles. After completing the full-text review of the 70 articles, 47 were included in this systematic review (Fig 1).

The 47 studies included in this review focused on 17 countries, ordered by number of publications: Nigeria (10 [14–23]), Ghana (7 [24–30]), Uganda (5 [31–35]), Liberia (4 [36–39]), the Democratic Republic of Congo (3 [17, 40, 41]), Ethiopia (3 [17, 42, 43]), Kenya (3 [44–46]), Sierra Leone (2 [47, 48]), Zimbabwe (2 [49, 50]), Angola (1 [17]), Botswana (1 [51]), Cameroon (1 [52]), Madagascar (1 [53]), Malawi (1 [54]), Tanzania (1 [17]), Togo (1 [17]), and Zambia (1 [55]). Four studies examined IDSR strategy on a regional level [56–59] (Table 1).

**Table 1:** Summary characteristics of included articles, listed by year of publication and then alphabetically by author.

No.	Author(s)	Ref No.	Country of Origin	Disease	Year	Level of Focus	Year Implemented	Priority Disease Detection/ Notification	Data Reporting	Data Management	Data analysis	Information Dissemination	Outbreak response, preparedness, & control	Laboratory	Personnel	Challenges with IDSR Implementation	Gaps requiring further studies
								N = 27	N = 39	N = 13	N = 12	N = 23	N = 20	N = 13	N = 21	N = 38	N = 13
**1**	Fall et al.	[9]	Regional	Multiple	2019	District	1998–2007		**X**			**X**			**X**	**X**	
**2**	Frimpong-Mansoh et al.	[30]	Ghana	TB	2019	District	2002		**X**	**X**	**X**	**X**					
**3**	Masiira et al.	[35]	Uganda	Multiple	2019	Healthcare workers, Health facility, District, National	2000		**X**	**X**	**X**	**X**	**X**	**X**	**X**	**X**	
**4**	Nagbe et al.	[38]	Liberia	Multiple	2019	Health facility, Community	2004	**X**	**X**	**X**	**X**	**X**	**X**	**X**	**X**	**X**	
**5**	Nagbe et al.	[39]	Liberia	Multiple	2019	County, District, Health facility	2004			**X**			**X**	**X**	**X**	**X**	
**6**	Nakiire et al.	[34]	Uganda	Multiple	2019	Healthcare workers	2000						**X**		**X**	**X**	
**7**	Njuguna et al.	[48]	Sierra Leone	Multiple	2019	District, Health facility	2003	**X**	**X**			**X**	**X**		**X**	**X**	
**8**	Curran et al.	[44]	Kenya	Cholera	2018	County, Healthcare workers	2005	**X**	**X**			**X**	**X**	**X**	**X**	**X**	
**9**	Jinadu et al.	[23]	Nigeria	Multiple	2018	Healthcare workers	2000	**X**	**X**						**X**	**X**	
**10**	Joseph Wu et al.	[54]	Malawi	Multiple	2018	Community, Health facility, District	2002	**X**	**X**					**X**		**X**	
**11**	Motlaleng et al.	[51]	Botswana	Malaria	2018	District	2001		**X**						**X**	**X**	**X**
**12**	Randriamiarana et al.	[53]	Madagascar	Multiple	2018	Region, Healthcare Facility	2007	**X**	**X**	**X**	**X**	**X**				**X**	
**13**	Toda et al.	[46]	Kenya	Multiple	2018	Health facility	2005	**X**	**X**			**X**			**X**	**X**	**X**
**14**	Uchenna et al.	[22]	Nigeria	AFP, measles	2018	State, LGA, Health facility	2000	**X**	**X**	**X**	**X**	**X**	**X**		**X**	**X**	**X**
**15**	Ashbaugh et al.	[41]	Democratic Republic of Congo	Ebola	2017	Health facility	2000	**X**	**X**								
**16**	Hamblion et al.	[37]	Liberia	Lassa Fever	2017	Multiple	2004		**X**	**X**			**X**	**X**			**X**
**17**	Jephcott et al.	[29]	Ghana	Emerging Infectious Diseases	2017	Community	2002	**X**				**X**				**X**	
**18**	Lakew et al.	[43]	Ethiopia	AFP	2017	Woreda (zone/reporting unit)	1998	**X**	**X**		**X**	**X**	**X**	**X**	**X**	**X**	
**19**	Mandyata et al.	[55]	Zambia	Multiple	2017	Healthcare worker	1998	**X**	**X**	**X**	**X**	**X**	**X**	**X**	**X**	**X**	
**20**	Muchena et al.	[49]	Zimbabwe	Malaria	2017	District	1998		**X**		**X**		**X**		**X**	**X**	
**21**	Mutsigiri-Murewanhema et al.	[50]	Zimbabwe	Maternal mortality	2017	District	1998		**X**			**X**				**X**	
**22**	Nass et al.	[18]	Nigeria	Neonatal Tetanus	2017	Health facility	2000	**X**	**X**							**X**	**X**
**23**	Nsubuga et al.	[33]	Uganda	Measles	2017	District (112 in total)	2000	**X**	**X**							**X**	
**24**	Wassilak et al.	[21]	Nigeria	AFP, Ebola	2017	District	2000						**X**				**X**
**25**	Adokiya et al.	[27]	Ghana	Multiple	2016	District (13 in the Upper East Region)	2002		**X**							**X**	**X**
**26**	Adokiya et al.	[24]	Ghana	Ebola	2016	Multiple	2002	**X**	**X**		**X**	**X**	**X**		**X**	**X**	
**27**	Benedetti et al.	[40]	Democratic Republic of the Congo	Multiple	2016	Health zones	2000	**X**					**X**				**X**
**28**	Mwatondo et al.	[45]	Kenya	Multiple	2016	Health facilities (within Nairobi county)	2005		**X**	**X**							**X**
**29**	Mwengee et al.	[17]	Angola, Democratic Republic of the Congo, Cote d’Ivoire, Ethiopia, Nigeria, Tanzania, and Togo	Polio/AFP	2016	National	1998–2000	**X**	**X**	**X**						**X**	
**30**	Ngwa et al.	[52]	Cameroon	Cholera	2016	National	2003		**X**	**X**	**X**	**X**	**X**	**X**		**X**	**X**
**31**	Poy et al.	[59]	Regional	Multiple	2016	Multiple	1998–2002	**X**	**X**	**X**		**X**			**X**		
**32**	Wesseh et al.	[36]	Liberia	Multiple	2016	Multiple	2004									**X**	
**33**	Adokiya et al.	[25]	Ghana	Multiple	2015	District (13 in the Upper East Region)	2002	**X**	**X**			**X**	**X**	**X**	**X**	**X**	
**34**	Adokiya et al.	[26]	Ghana	Multiple	2015	Health facility	2002	**X**	**X**		**X**	**X**	**X**		**X**	**X**	
**35**	Issah et al.	[28]	Ghana	Ebola	2015	State/Region	2002	**X**	**X**			**X**	**X**		**X**	**X**	
**36**	Lar et al.	[16]	Nigeria	Multiple	2015	LGA	2000		**X**			**X**					
**37**	Nguku et al.	[58]	Regional	Multiple	2015	Health facility, District, National	1998–2002							**X**		**X**	
**38**	Lafond et al.	[15]	Nigeria	Avian influenza and other priority diseases	2014	Healthcare worker	2000	**X**	**X**			**X**				**X**	**X**
**39**	Mbondji et al.	[57]	Multiple	Multiple	2014	National	1998–2002		**X**						**X**	**X**	
**40**	Borchert et al.	[31]	Uganda	Ebola; Cholera; MDR-TB	2013	District	2000	**X**	**X**				**X**	**X**			
**41**	Fatiregun et al.	[14]	Nigeria	Cholera	2013	District	2000	**X**	**X**							**X**	
**42**	Kasolo et al.	[56]	Regional	Multiple	2013	Multiple	1998–2002		**X**							**X**	
**43**	Kebede et al.	[47]	Sierra Leone	Influenza	2013	Health facility	2003	**X**								**X**	**X**
**44**	Lukwago et al.	[32]	Uganda	Multiple	2013	District, National	2000		**X**	**X**	**X**	**X**	**X**	**X**		**X**	
**45**	Nnebue et al.	[20]	Nigeria	Multiple	2013	State	2000	**X**	**X**			**X**				**X**	
**46**	Nnebue et al.	[19]	Nigeria	Multiple	2012	Healthcare worker, Health facility, Local Government Area	2000	**X**	**X**						**X**	**X**	
**47**	Jima et al.	[42]	Ethiopia	Malaria	2012	Woreda (zone/reporting unit)	1998		**X**							**X**	**X**

The included studies are presented and organized in Table 1 by year of publication and then author last name. Additional information presented includes country of focus, diseases of focus, and the IDSR thematic areas discussed within each study. The thematic areas most commonly reported on were data reporting, challenges with IDSR implementation, and priority disease detection and notification.

**Fig 2:**
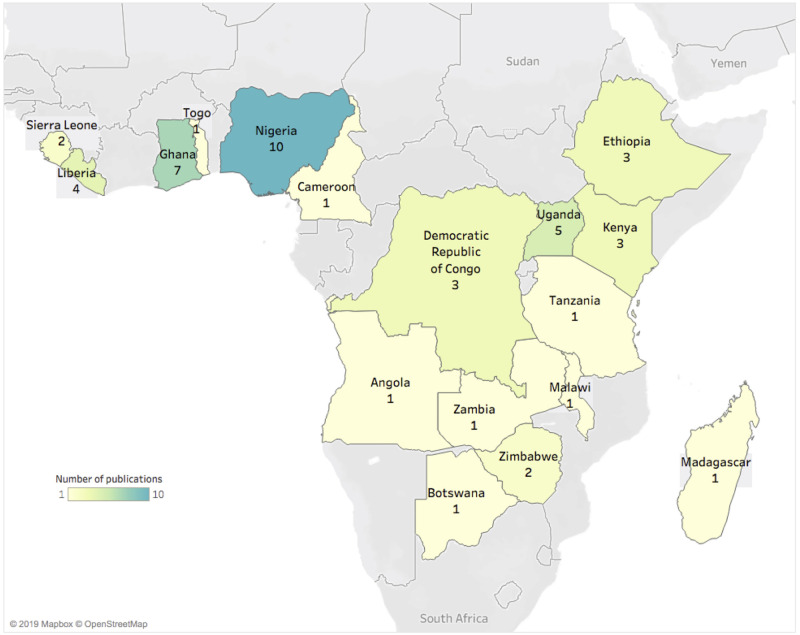
Geographic distribution of included studies. Number of publications discussing IDSR among the 17 included countries in the African Region. The countries with the most publications were Nigeria, Ghana, and Uganda.

Based on studies in this review, the number of peer-reviewed articles discussing IDSR remains low. Of 47 Member States in the African region, only 35% (n = 17) are represented in this systematic review. While some countries in the region are more regularly producing publications, as demonstrated by the literature focusing on Nigeria, Ghana, and Uganda (Fig 2), more than half of Member States are not producing any scientific literature on IDSR activities.

**Fig 3:**
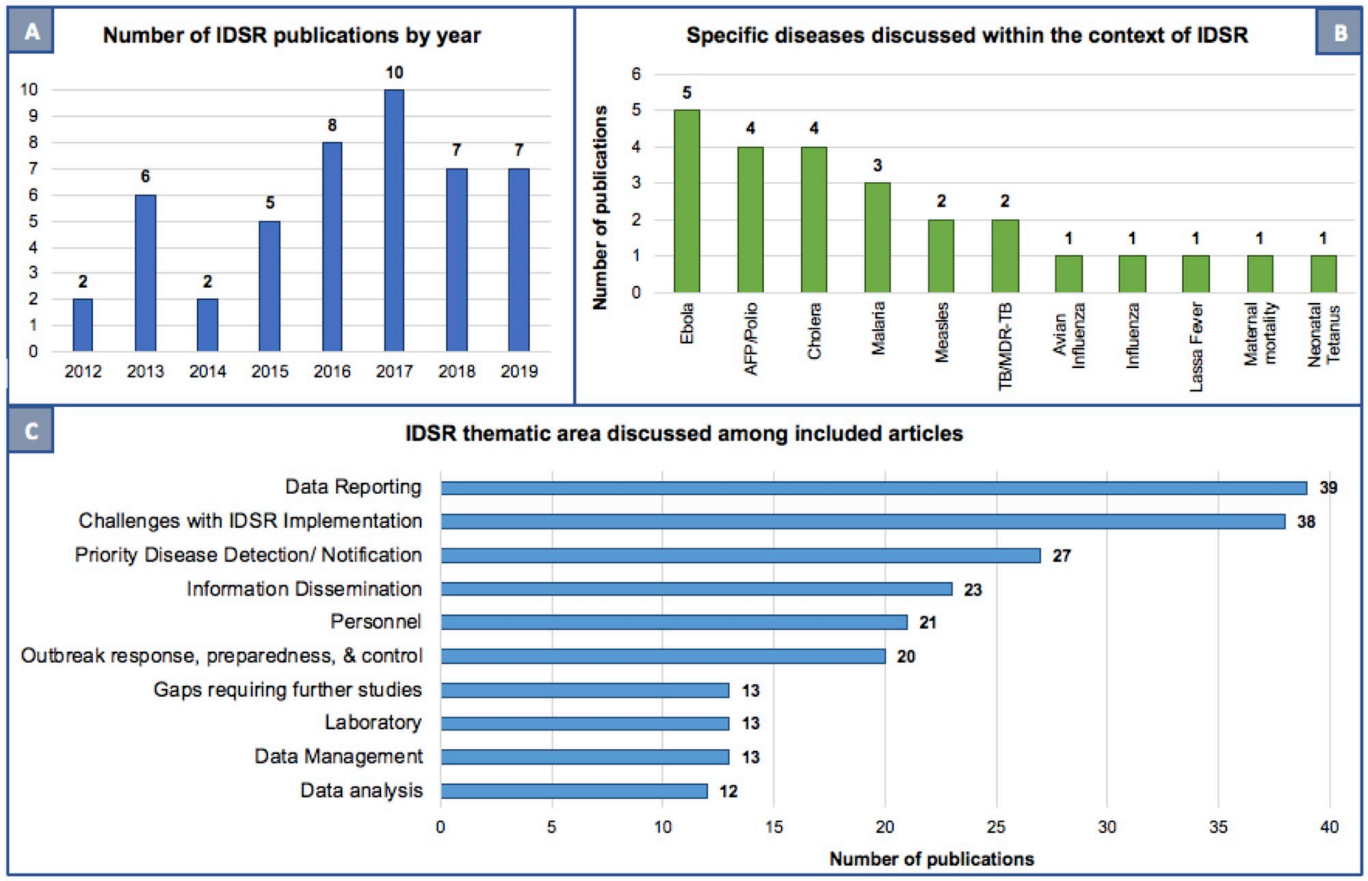
Publication year, specific diseases, and key IDSR core and support functions discussed in among included literature. Number of IDSR publications by year included in this analysis (panel A), specific diseases discussed among the included articles (panel B), and IDSR core and support functions represented (panel C).

The average time from initial IDSR implementation to study publication was 15 years. Of the years captured within this review (latter half of 2012 through November 2019), most articles were published in 2017 (n = 10, 21.3%), followed by 2016 (n = 8, 17%) (Fig 3, panel A). The number of publications in the last four years (n = 32) is more than double the number of studies in the preceding four years (n = 15). Five studies (10.6%) evaluated IDSR performance at the national and county or state level, 13 (27.7%) at the district level, 8 (17%) at the health facility level, four (8.5%) among healthcare workers and one at the community level. Several articles assessed IDSR in the context of disease-specific surveillance (Fig 3, panel B). Five (10.6%) discussed Ebola, four (8.5%) each on acute flaccid paralysis (AFP)/poliomyelitis and cholera, three (6.4%) on malaria, two each (4.3%) on measles and tuberculosis, and one each on avian influenza, seasonal influenza, Lassa fever, maternal mortality, and neonatal tetanus. Thirty (63.8%) of the reviewed studies focused on and discussed multiple diseases. Study characteristics are further summarized in Table 1.

The three most common IDSR core and support functions discussed were data reporting (83%, n = 39), priority disease detection and notification (57%, n = 27) and information dissemination (52%, n = 23) (Table 2 and Fig 3, panel C). Other commonly discussed core functions included outbreak response, preparedness, and control (42.6%, n = 20), personnel and staff training (44.7%, n = 21), laboratory capacity (27.7%, n = 13), and data management (27.7%, n = 13). Data analysis was the thematic area discussed least, appearing in only a quarter of included studies (25.5%, n = 12). Over 80% of included studies also discussed challenges with IDSR implementation (81%, n = 38).

**Table 2:** Key findings by core and support functions assessed under IDSR.

Thematic area	N (%) of studies	Key findings	
Priority disease detection/ notification	27 (57)	Common challenge(s)	Underreporting of certain diseases through IDSR mechanisms. Specific examples were measles [22] and neonatal tetanus [18].
			Multiple surveillance systems.
			Confusion on correct forms to use and how to use them [15].
			Gap in incorporating emerging infectious disease surveillance at the community level.
		Highlighted success(es)	Cholera and other epidemic-prone diseases were routinely reported (Ghana [25]).
			Successful merger of influenza surveillance with existing IDSR mechanisms (Sierra Leone [47]).
Data reporting	39 (83)	Common challenge(s)	Lack of availability of forms.
			Inconsistencies between weekly and monthly disease totals.
			Discrepancies between data reported through IDSR systems and disease-specific systems.
			Reporting deadlines were poorly understood and varied by states, districts and even facilities within a district.
		Highlighted success(es)	Using e-IDSR, the proportion of suspected outbreaks and public health events detected through the IDSR system was 96% (*n* = 87) in 2016 and 100% (*n* = 85) in 2017 (Sierra Leone [48]).
			Data reporting for malaria found to be efficient in both Ethiopia [42] and Botswana [51].
			Designated focal person increased the odds of adequate reporting (Kenya [45]).
Data management	13 (28)	Common challenge(s)	Poor and inadequate documentation and data management.
			Falsification of data by health personnel (specific example in Nigeria [22]).
		Highlighted success(es)	Enhanced capacity for data management following a baseline IDSR assessment (Uganda [30]).
			AFP surveillance provided a functional infrastructure, trained personnel, and other resources used to implement IDSR [17].
Data analysis	12 (26)	Common challenge(s)	Lack of data analysis or interpretation at the health facility and health district levels.
			Data analysis and utilization not taken seriously.
		Highlighted success(es)	AFP and measles surveillance indicators were regularly calculated and monitored in several health districts (Ethiopia [43]).
			Evidence of improvement in data analysis at health facilities and monitoring of disease trends at the district level (Uganda [32]).
Information dissemination	23 (49)	Common challenge(s)	Lack of regular feedback after reporting.
			Frustration with constant sharing of case counts and data to county level without getting updates in return.
		Highlighted success(es)	Most common information dissemination reported within the IDSR system was the release of health bulletins from the national level.
Outbreak response preparedness and control	20 (43)	Common challenge(s)	Effectiveness hampered due to mainly poor technical expertise, weak laboratory infrastructures, limited transport capacities, and a lack of pre-positioned emergency stock supplies (drugs and other essentials).
			Limited access to budgets for epidemic response.
			Response efforts guided by ad hoc emergency committees.
			Absent or incomplete documentation of outbreak management limited evaluations and led to poor institutional learning.
			Preparedness for immediate investigation of suspected outbreaks was weak at the health facility and health district.
Laboratory capacity	13 (28)	Common challenge(s)	Hamstrung by inadequate staffing (in number and skill of the available staff).
			Lack of availability or shortages of laboratory supplies, equipment, and servicing both in quantity and quality.
			Inadequate financial resources, lack of equipment, reagents, and training in sample collection, limited storage, transport, compounded by inadequate lab technicians, delayed case and outbreak confirmation.
		Highlighted success(es)	Joint US CDC and Uganda MOH project demonstrated success in improving lab capacity and referral network [31].
Personnel and staff training	21 (45)	Common challenge(s)	Supervision and feedback low at the peripheral levels.
			Major determinants of staff motivation include lack of feedback and supportive supervision.
			Surveillance training only made available to certain individuals, and not all identified individuals can afford to take part.

Common challenges and highlighted successes by core and support functions as assessed under IDSR.

Table 2 summarizes the key findings regarding common challenges and highlighted successes by core and support functions as assessed under IDSR. Outbreak response, preparedness, and control along with personnel were the only thematic areas without highlighted successes (Table 2).

### Data reporting

Thirty-nine studies (83%) reported challenges with data reporting, with effectiveness varying by country and specified diseases. Commonly reported issues included lack of availability of forms and inconsistencies between weekly and monthly disease totals. Additionally, four studies reported discrepancies between data reported through IDSR strategy and disease-specific systems. Uchenna et al. found that both measles and cholera were underreported in Nigeria [22]. Nass et al. found neonatal tetanus to be underreported and ineffective in the current IDSR strategy [18]. In contrast, some studies also reported successes. Jima et al. and Motlaleng et al. both found the data reporting for malaria to be efficient in Ethiopia [42] and Botswana [51], respectively. In Kenya, having a designated surveillance focal person at health care facilities increased the odds of adequate reporting compared to facilities with no designated focal person [45].

### Priority disease detection

The quality and effectiveness of priority disease detection varied by country and disease. For example, one study in Nigeria found an underreporting of observed measles cases [22] and another found that only 33% of identified neonatal tetanus cases identified through active surveillance were reported through IDSR mechanisms [18]. In Ghana, there were concerns about the establishment of what seemed like a second surveillance system for Ebola [28] and gaps incorporating emerging infectious diseases, especially at the community level [29]. Another Ebola-focused article indicated that only half of key respondents described the existing surveillance system as functional [24]. In terms of success, cholera cases and other epidemic-prone diseases were routinely reported in Ghana, though there were some technical and organizational challenges [25]. Additionally, Sierra Leone successfully merged their influenza surveillance with previously existing mechanisms for implementation of IDSR back in 2012 [47].

### Information dissemination

The most common information dissemination reported within the IDSR strategy was the release of epidemiological bulletins from the national level. These national bulletins were often the only feedback mechanism for the culmination of information reported up from the various levels within IDSR. In Ghana, key informants reported that no real feedback to the periphery existed, with IDSR data compiled at the regional level in the form of an excel spreadsheet and sent back to the district officers but not to the health facilities [25]. Feedback to these health facilities was only communicated during unit head meetings or at the bi-annual review meetings. Additionally, when reviewing the IDSR strategy in the context of Ebola, respondents indicated that apart from investigations by phone in case of rumors, feedback and information on EVD surveillance to health facilities only occurred during monthly unit head meetings or at bi-annual/annual review meetings [24]. Similarly, when involved in cholera reporting, health care workers in Kenya expressed frustration at constantly sharing case counts to county representatives without receiving feedback in return [44].

### Outbreak response, preparedness, and control

In terms of preparedness, key informants in Ghana indicated that the availability of emergency stocks of medicines and other essential supplies were inadequate in the districts, and some of the challenges were partly attributed to the district assemblies’ failure to provide budgetary and logistics support [25]. Response efforts were found to be guided by ad hoc emergency committees, which consequently delayed the necessary response actions significantly. Absent or incomplete documentation of outbreak management and limited evaluations led to poor institutional learning. In Cameroon, preparedness for immediate investigation of suspected outbreaks was weak at the health facility and health district but strong at the regional level [52]. Even following the lessons learned and subsequent investments in the health systems infrastructure following the 2014–2016 EVD outbreak in Liberia, there was late recognition of a Lassa fever outbreak that occurred in 2016. Twelve suspected cases were notified but not investigated, and personal protective equipment supplies were not sufficient for an extended outbreak [37].

## Discussion

Though lessons were learned from recent epidemics and outbreaks, several key challenges continue to hinder the effective implementation of the IDSR strategy in the African region. Unsustainable financial resources, lack of coordination, inadequate training and turnover of peripheral staff, erratic feedback, inadequate supervision from the next level, weak laboratory capacities coupled with unavailability of job aids (e.g. case definitions, reporting forms), and poor availability of communication and transport systems are all challenges impeding IDSR implementation that have been reported on previously [8]. Additionally, there were barriers to effective IDSR implementation identified across IDSR core and support functions assessed in this review: priority disease detection; data reporting, management, and analysis; information dissemination; laboratory functionality; and staff training (Table 2).

### Common challenges with effective IDSR implementation

The most commonly reported challenges surrounded data reporting. There were issues with data accuracy and zero reporting, incomplete data, and reporting delays. Moreover, there were documented instances of underreporting for certain diseases, which led to concerns about the effects of underreporting on health budgets. Unavailability of reporting forms was also a common complaint. Lack of resources (financial, material, transportation, and inadequate staff numbers) was the second most common identified challenge. Other concerns included ineffective priority disease detection, lack of knowledge around case definitions, terms of reference for staff and personnel, surveillance procedures, and the lack of documentation of preparedness and response plans. While only one included study discussed documented data falsification, any form of incentivized reporting can lead to perverse effects in either direction: possible overreporting to meet higher targets, and possible underreporting to meet lower targets.

Some of the specific concerns raised transcended particular diseases, including the use of parallel data collection systems (case-based surveillance vs. IDSR) and how reporting requirements put additional burden on the health care facility staff. These additional burdens are further compounded by the challenges with information feedback. It is difficult to embrace the importance of timely and complete reporting when it isn’t always clear how the reported data is used to guide public health action. Additionally, those frequently working with polio surveillance stressed the need to streamline existing structures and to build on existing capacity, particularly in remote areas. In the Democratic Republic of the Congo, teams from Médecins Sans Frontières demonstrated that combining resources and priorities when emergency interventions were used was an opportunity to strengthen and improve disease surveillance [40]. One of the studies in Ghana highlighted that IDSR was developed with specific priority diseases in mind, therefore indicating there is room to improve on incorporating surveillance for emerging infectious diseases into the existing system within the country [29]. The strength of laboratory networks in the region has improved in recent years, but more work is still needed. These laboratories and networks have been stymied by inadequate staffing (in number and skill of the available staff) and unavailability or shortages of laboratory supplies, equipment, and servicing both in quantity and quality [58]. This has resulted in regular breakdown and frequent interruption of services, challenges in developing robust systems, and difficulty training the workforce to operate public health laboratory networks [58]. Another common finding that was reported previously [8] was that supervision, particularly at the peripheral levels, was poor.

The issues surrounding data reporting are concerning, as this remains a fundamental component of successful surveillance systems [60]. Decisions in terms of outbreak response, preparedness, and resource distribution are made based on available data and without it, any analyses performed provide an incomplete picture. These analytics and the resulting outputs are central tenants of a surveillance pillar of any outbreak response, yet the resources and capacities to ensure data availability and quality are often limited [61]. Limiting factors include lack of resources and trained personnel, both of which were also frequently discussed challenges of IDSR implementation. Effective response to such outbreaks relies on timely intervention, ideally informed by all available sources of data [60, 61], yet ensuring the required data is collected and reported in a timely manner requires sufficient resources and a trained workforce.

### Highlighted successes

In terms of data and reporting, cholera and other epidemic-prone diseases were routinely reported in Ghana [25]. Malaria reporting was efficient in both Ethiopia [42] and Botswana [51]. In Kenya, having a designated focal person increased the odds of adequate reporting [45]. Utilizing existing AFP surveillance mechanisms provided functional infrastructure, trained personnel, and other resources to more efficiently implement IDSR [17]. In Sierra Leone, the successful merger of influenza surveillance with existing IDSR mechanisms led to an increase in influenza reporting [47]. Uganda demonstrated an enhanced capacity for data management following a baseline IDSR assessment [32]. In Ethiopia, AFP and measles surveillance indicators were regularly calculated and monitored in several health districts [43]. Evidence of improvement in data analysis at health facilities and monitoring of disease trends were seen at the district level over time in Uganda [32]. Across the board, the most common information dissemination reported within the IDSR strategy was the release of epidemiological bulletins from the national level.

The examples of utilizing existing surveillance systems highlight a potential mechanism of streamlining and improving efficient IDSR implementation as this infrastructure has already been used in efforts to increase access to healthcare [62]. The reduction of redundancy, such as the merging of influenza surveillance with IDSR, may also lead to better reporting. The spotlighted successes represent ideas for improvement and can serve as examples for countries looking to enhance their implementation of IDSR.

### Limitations

There are limitations of this systematic review. The literature search strategy was intentionally restricted to only include peer-reviewed literature, as these IDSR information products were the focus of this review. We did, therefore, not include country-specific IDSR technical guidelines, reports written by respective Ministries of Health or WHO country offices, or other grey literature. Additionally, only articles written in English and French were included. Whilst these are the two main working languages in the region, Portuguese and Spanish are also used in some Member States. While articles in other languages were not specifically excluded for that reason, it is possible that articles written in other languages were not identified by the search strategy employed.

Further, the search strategy specifically focused on integrated disease surveillance and response. It is possible that articles discussing individual portions of the strategy or specific diseases within the IDSR framework without linking them back to the broader strategy may not have been captured by the predetermined search terms. Lastly, the involvement of WHO in this review as one of the entities responsible for the development and implementation of IDSR could be seen as a possible source of analytical bias. However, multiple coauthors were not affiliated with WHO and, those affiliated with WHO were not involved in the IDSR development process. To minimize the potential for bias, a rigorous search plan and process was developed and agreed upon. Furthermore, it was the institutional familiarity with the IDSR system which highlighted notable gaps during the 2018 revision of the technical guidelines, prompting this review of IDSR implementation.

### Future considerations

The IDSR strategy provides a framework for strengthening surveillance, response, and laboratory capacities as required by the 2005 revised IHR [56]. Since initial development of IDSR in 1998, countries in the WHO African region have adopted the strategy over the years and developed their own national IDSR guidelines tailored to their diseases of concern. As such, the status of IDSR implementation has reached varying levels in countries across the African region. Despite these varying levels of implementation, the IDSR system has been leveraged in response to the ongoing COVID-19 pandemic and has helped prolong the containment phase of COVID-19 in many countries across the continent [63]. Of particular relevance, the IDSR framework has facilitated case-based and syndromic surveillance of many conditions, including influenza-like illness and severe acute respiratory illness. This framework has allowed for intensive surveillance and case finding, providing an entry point for identifying, characterizing, and responding to community transmission of COVID-19 [63].

Investments in electronic IDSR (e-IDSR) systems or other digital disease surveillance and reporting tools may serve as a potential solution to some of the noted priority disease detection, reporting, and data management challenges. Sierra Leone recently became the first country in the WHO African region to fully transition from a paper-based to web-based electronic platform for disease surveillance [64]. While appealing, this would require sustained investments in internet infrastructure, as e-IDSR is dependent on strong internet connections and is therefore hampered by poor internet connectivity, common in remote areas of the region [48]. Internet connectivity can be challenging in remote locations, however additional digital tools such as Go.Data and EWARS may have some functionality offline by means of local storage, however, an adequate internet connection is often required to upload and submit any data collected on the platforms [65]. Improvements in data collection, management, reporting, and dissemination during outbreaks is needed to ensure accurate understanding, easy scale up, and effective analyses to inform response actions, and bring a rapid end to disease transmission.

Furthermore, in countries that have adopted the IDSR strategy, one of the key requirements is developing and dissemination of information products (including scientific articles) to inform decision making by policy makers. In the review conducted by Phalkey et al. [8], 16 countries from the WHO African region were represented in the included literature. Seven years later, only one additional country has published peer-reviewed IDSR literature. As this remains a weak point, future efforts should focus on information dissemination. Additionally, over 30% of the included studies were published in region-specific journals. Moving forward, it would be beneficial to consider publishing in journals that cover a broader geographic area to expand the global reach of IDSR-related literature. Lastly, dissemination of information back down to the level of collection—for example, the health facilities or heath districts—is also imperative for clear, effective supervision and communication about how collected data is used to guide public health actions. Strengthening the feedback mechanisms within information dissemination allows for continuous monitoring and adaptation and may assist with instilling a sense of ownership and collaboration in the IDSR process.

### Public health implications

Issues identified with the implementation of IDSR have remained consistent over time. As such, it is clear that to strengthen surveillance and response systems in Member States, improvement in surveillance and response actions improvement in coordination, financial resources, staff training and available job aids, supervisor feedback, laboratory capacity, communication systems, and transportation networks is imperative. Robust public health systems require effective surveillance and adequate outbreak response capacity, along with trained and informed personnel. Infectious disease outbreaks have the potential to harm national and regional economies [9], as the COVID-19 pandemic has also clearly demonstrated, and as such, it is in the African region’s best interest to be able to respond efficiently to events to help mitigate their devastation.

## Conclusions

The International Health Regulations (2005) require fully functioning disease surveillance systems for preparedness planning and broader global health security. IDSR is a comprehensive, evidence-based strategy for strengthening national public health surveillance and response systems at all levels [9], yet the implementation of this strategy remains incomplete. The associated challenges that have plagued IDSR implementation since the first edition of the technical guidelines were released are not novel, however adequately addressing them requires sustained investments in stronger national public health capabilities, infrastructure and surveillance processes [54, 60, 61, 65, 66].

## Supporting information

S1 Appendix Priority disease, conditions, and events for Integrated Disease Surveillance and Response– 2010 [1]. The adoption of the revised International Health Regulations (IHR) in 2005 further demanded a revision of the 1998 IDSR TG in 2010 [2]. The revision of the IDSR technical guidelines in 2010, following the adoption of the revised International Health Regulations (IHR) in 2005, proposed an alteration to the four categories of priority diseases, conditions, and events for Integrated Disease Surveillance and Response (IDSR) to epidemic prone diseases, diseases targeted for eradication or elimination, other major diseases, events, or conditions of public health importance, and diseases or events of international concern [2, 7].
(TIF)

S2 Appendix Priority disease, conditions and events for Integrated Disease Surveillance and Response– 2018 [10]. The third revision of the IDSR technical guidelines occurred in 2019 and aimed to align the IDSR framework with the introduction of the Regional Strategy for Health Security and Emergencies 2016–2020 in the WHO African region [12]. This version incorporates new information technologies such as mobile phone networks, increased broadband internet connectivity and electronic surveillance systems.
(TIF)

S3 Appendix Search strategies. (DOCX)

S1 Checklist PRISMA 2009 checklist. (DOC)
