# Supplementary figures and images for: Systematic review of Integrated Disease Surveillance and Response (IDSR) implementation in the African region
Source: PLoS One. 2021 Feb 25;16(2):e0245457. doi: 10.1371/journal.pone.0245457 (PMC7906422; doi:10.1371/journal.pone.0245457)

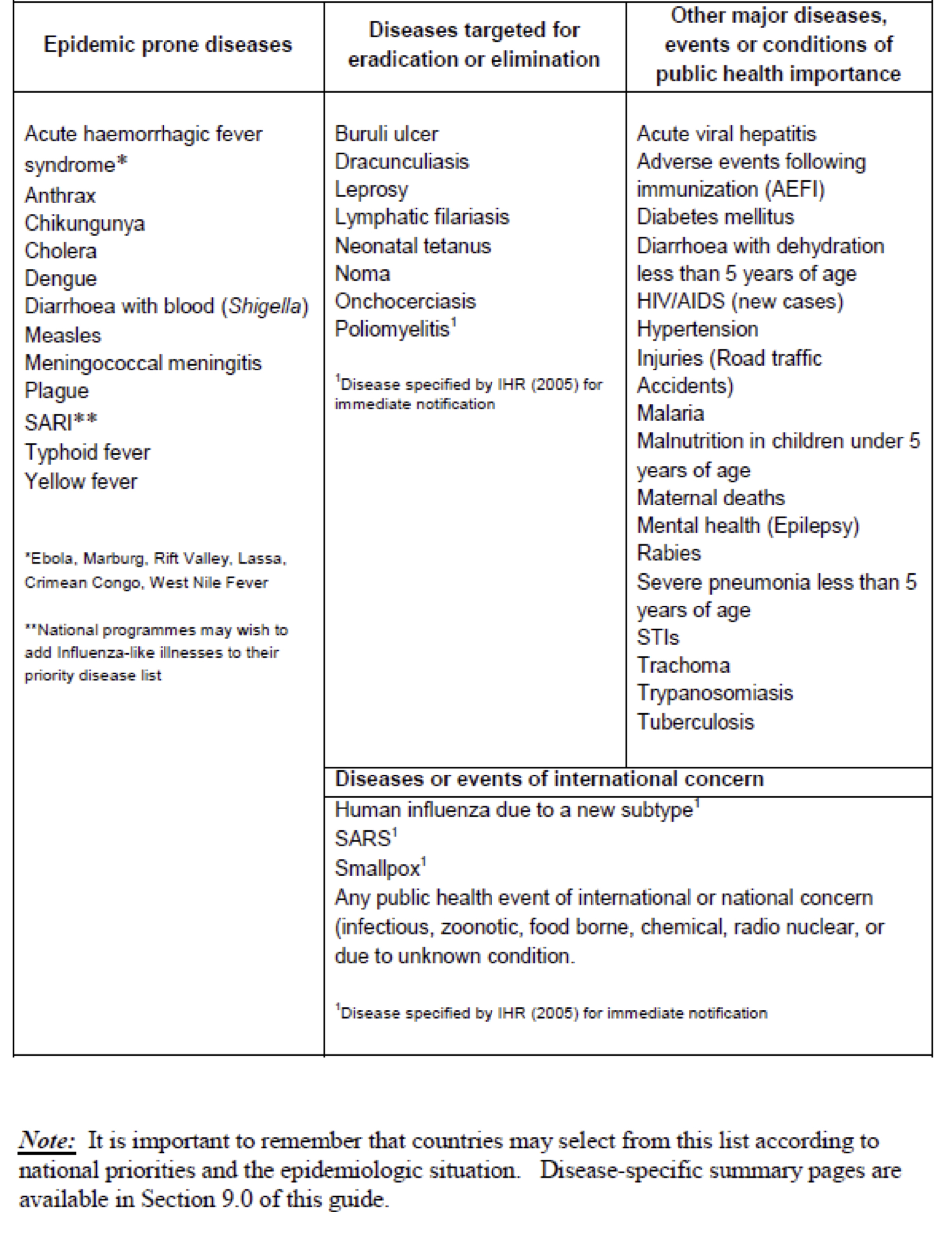

Supplement: S1 Appendix — The adoption of the revised International Health Regulations (IHR) in 2005 further demanded a revision of the 1998 IDSR TG in 2010 [2]. The revision of the IDSR technical guidelines in 2010, following the adoption of the revised International Health Regulations (IHR) in 2005, proposed an alteration to the four categories of priority diseases, conditions, and events for Integrated Disease Surveillance and Response (IDSR) to epidemic prone diseases, diseases targeted for eradication or elimination, other major diseases, events, or conditions of public health importance, and diseases or events of international concern [2, 7]. (TIF) [file pone.0245457.s001.tif]

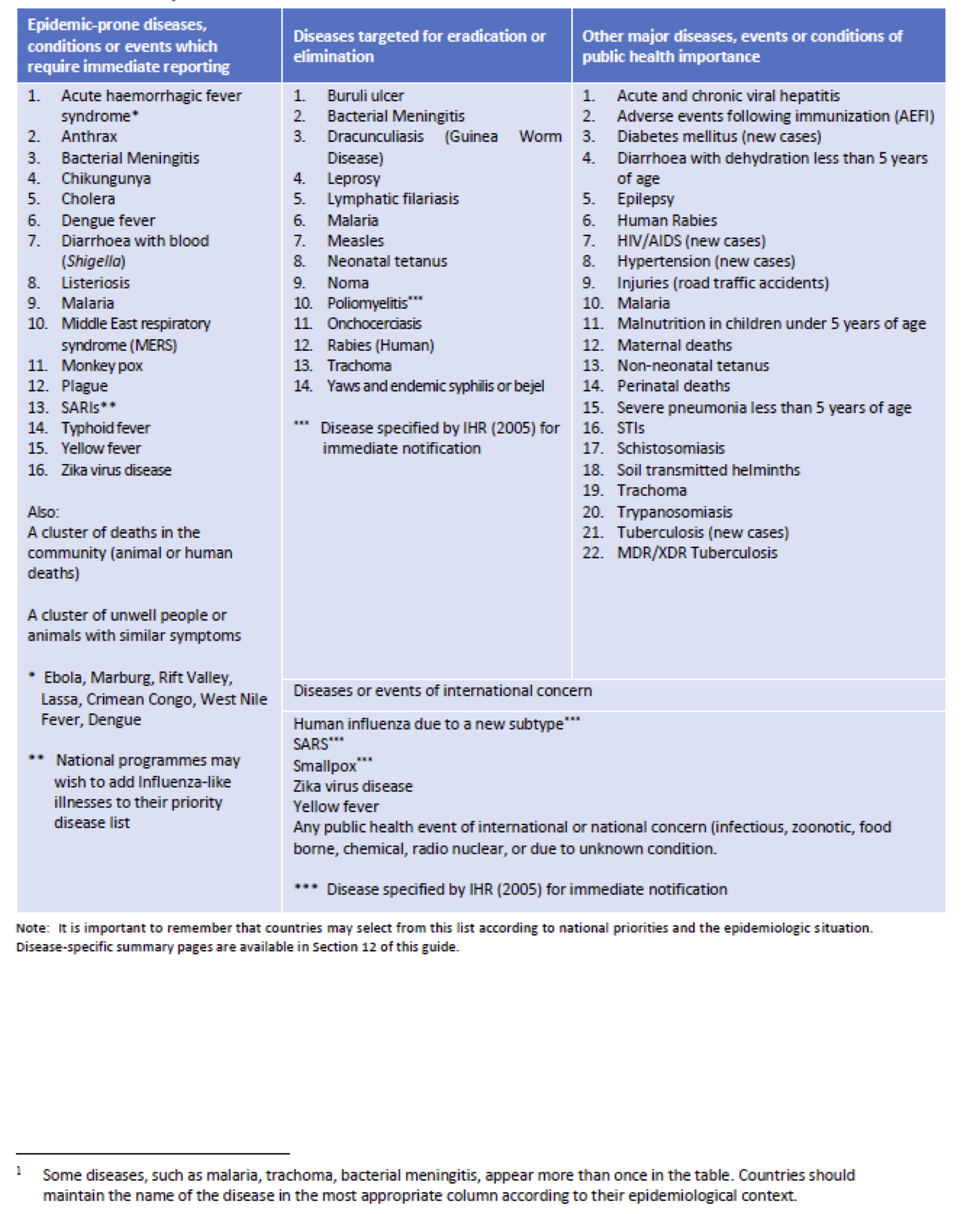

Supplement: S2 Appendix — The third revision of the IDSR technical guidelines occurred in 2019 and aimed to align the IDSR framework with the introduction of the Regional Strategy for Health Security and Emergencies 2016–2020 in the WHO African region [12]. This version incorporates new information technologies such as mobile phone networks, increased broadband internet connectivity and electronic surveillance systems. (TIF) [file pone.0245457.s002.tif]
