# Supplementary material for: Systematic review of Integrated Disease Surveillance and Response (IDSR) implementation in the African region
Source: PLoS One. 2021 Feb 25;16(2):e0245457. doi: 10.1371/journal.pone.0245457 (PMC7906422; doi:10.1371/journal.pone.0245457)
Supplement: S3 Appendix — (DOCX) [file pone.0245457.s003.docx]

**S3 Appendix. Search strategies.**

PubMed search strategy:

English: ("Integrated Disease Surveillance and Response"[tiab]) OR (IDSR[tiab]) OR ("IDSR Implementation"[tiab]) OR ("IDSR Evaluation"[tiab]), date range: 2012 – 2019

Français: ("Surveillance Intégrée de la maladie et la riposte"[tiab]) OR ("SIMR"[tiab]) OR "Mise en œurve de la SIMR"[tiab]) OR ("Évaluation de la SIMR"[tiab]), date range: 2012 - 2019

Web of Science search strategy:

English: TS=((Integrated Disease Surveillance and Response) OR (IDSR) OR (IDSR Implementation) OR (IDSR Evaluation)); date range: 2012-2019

Français: TS=((Surveillance Intégrée de la maladie et la riposte) OR (SIMR) OR (Mise en œurve de la SIMR) OR (Évaluation de la SIMR)); date range: 2012-2019
